# When the Urine Bag Turns Purple: A Benign Manifestation in a Chronically Catheterized Elderly Woman

**DOI:** 10.7759/cureus.96209

**Published:** 2025-11-06

**Authors:** Zohre Alkan, Sibel Altunışık Toplu

**Affiliations:** 1 Home Health Care Unit, Pütürge State Hospital, Malatya, TUR; 2 Infectious Diseases, İnönü University, Malatya, TUR

**Keywords:** asymptomatic, catheter-associated urinary tract infection, elderly patient, purple urine bag syndrome, urinary catheter, urinary discoloration

## Abstract

Purple urine bag syndrome (PUBS) is a visually alarming but generally benign phenomenon observed in chronically catheterized patients. It occurs due to bacterial metabolism of tryptophan derivatives into indigo and indirubin pigments, which stain the catheter tubing and urine collection bag. We report the case of an 87-year-old woman with chronic kidney disease (CKD) who developed sudden purple discoloration of her urine drainage bag during home care follow-up. Laboratory evaluation revealed alkaline urine, leukocyte esterase positivity, mild proteinuria, and elevated creatinine. No urine culture result was available at the time of assessment. Based on urinalysis findings, particularly alkaline pH and pyuria, a catheter-associated asymptomatic condition was suspected. Following catheter and bag replacement, hydration, and observation without empiric antibiotics, the discoloration resolved completely. This case highlights that although PUBS can be visually distressing, it often represents a benign, asymptomatic phenomenon in catheterized elderly patients. Awareness of PUBS can help avoid unnecessary antibiotic use in elderly catheterized patients.

## Introduction

Purple urine bag syndrome (PUBS) is an uncommon but well-recognized condition associated with indwelling urinary catheters. First described in 1978, it results from bacterial enzymatic conversion of indoxyl sulfate (a tryptophan metabolite) into indigo (blue) and indirubin (red) pigments, combining to produce a striking purple color [1,2]. Common risk factors include advanced age, female sex, chronic kidney disease (CKD), constipation, prolonged catheterization, and alkaline urine [3]. Although the discoloration often causes panic among healthcare workers and families, PUBS is usually benign and reversible. Recent evidence indicates that indwelling urinary catheters remain common in long-term care settings, with a systematic review reporting a median prevalence of 7.3% among nursing home residents [4]. However, it may coexist with bacteriuria or catheter-associated urinary tract infection (CA-UTI) in some cases [5,6]. Awareness of this entity prevents unnecessary alarm and guides appropriate management.

## Case presentation

**Figure 1:**
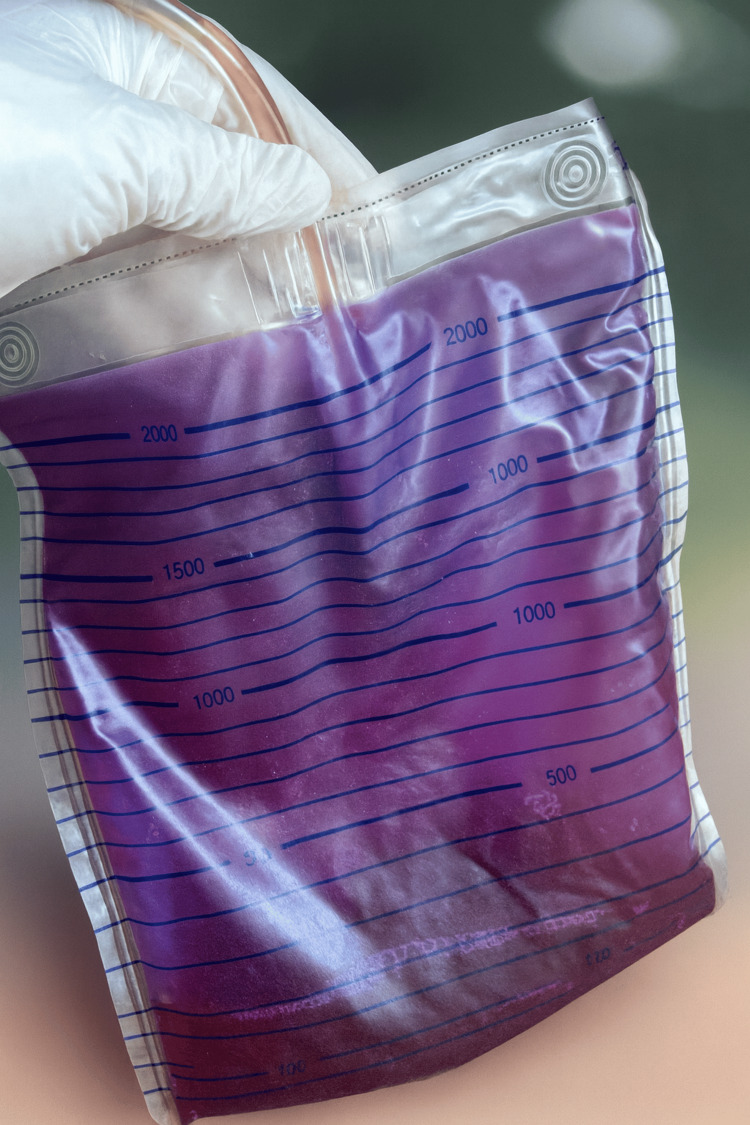
Purple discoloration of the urine collection bag and catheter tubing in our elderly, chronically catheterized patient. The striking violet hue resulted from pigment deposition (indigo and indirubin) associated with alkaline urine and asymptomatic leukocyturia. The patient remained afebrile and clinically stable, and the discoloration resolved within 48 hours following catheter and bag replacement.

An 87-year-old woman with a history of hypertension and stage 3-4 CKD was followed by home care services with a long-term Foley catheter. She required a chronic Foley catheter due to impaired mobility and urinary retention, and the catheter was being changed monthly during routine home healthcare visits. Caregivers noticed a sudden purple discoloration of the urine bag and tubing (Figure 1). On examination, the patient was afebrile and hemodynamically stable and exhibited no dysuria, flank pain, or systemic symptoms suggestive of UTI.

Laboratory evaluation demonstrated elevated blood glucose (266 mg/dL) and serum creatinine (2.29 mg/dL) with a decreased estimated glomerular filtration rate (eGFR; 21 mL/min/1.73 m²). Urinalysis showed alkaline urine (pH 7.5-8.5), leukocyte esterase 2+, mild proteinuria (1+), and seven to eight white blood cells per high-power field (HPF), but nitrite was negative (Table 1). A urine culture result was not available at the time of assessment, as the patient remained asymptomatic without clinical signs of infection. Given these findings, catheter-associated asymptomatic bacteriuria and metabolic imbalance secondary to CKD were considered.

**Table 1:** The patient's laboratory findings with reference ranges

Parameter	Result	Reference Range
Glucose	266 mg/dL	74–106 mg/dL
Creatinine	2.29 mg/dL	0.6–1.3 mg/dL
Estimated glomerular filtration rate (eGFR)	21 mL/min/1.73 m²	>90 mL/min/1.73 m²
Albumin	3.3 g/dL	3.5–5.0 g/dL
Potassium	5.32 mmol/L	3.5–5.1 mmol/L
Hemoglobin	9.8 g/dL	12–16 g/dL
WBC	7.64 ×10³/µL (62.7% neutrophils)	4–10 ×10³/µL
Urine pH	7.5–8.5	4.5–8.0
Leukocyte esterase	2+	Negative
Protein	1+	Negative
Nitrite	Negative	Negative
WBC (urine)	7–8 /HPF	0–5 /HPF
Specific gravity	1.020	1.005–1.030

Because of the elevated glucose and renal parameters, the patient was referred to the internal medicine outpatient clinic for further evaluation of glycemic control and renal function. At the same time, the Foley catheter and urine drainage bag were replaced under aseptic conditions, and adequate hydration was maintained. No empiric antibiotics were administered since there were no signs of infection.

Over the following 48 hours, the striking purple discoloration gradually disappeared, and the urine became clear. The patient remained afebrile and clinically stable throughout the observation period. At the three-week follow-up, there was no recurrence of discoloration. This improvement supported the interpretation that catheter and bag replacement, rather than antimicrobial therapy, was the key intervention leading to resolution.

## Discussion

PUBS is a rare manifestation of bacterial metabolism within the urinary tract. The indoxyl sulfate produced from dietary tryptophan in the liver is excreted into urine, where bacteria expressing indoxyl sulfatase/phosphatase, such as *Providencia*, *Proteus*, *Klebsiella*, *Escherichia coli*, and *Morganella *species, convert it into indigo and indirubin pigments [5,6]. These pigments adhere to polyvinyl chloride (PVC) catheter material, yielding the purple appearance. Alkaline urine and decreased renal clearance facilitate pigment accumulation [7]. Recent reports emphasize that PUBS occurs mainly in elderly, catheterized women with multiple comorbidities [8,9]. Most cases resolve with catheter replacement and hydration, with antibiotics required only for symptomatic infection [6,7]. Differential diagnoses for purple or discolored urine include hematuria, myoglobinuria, porphyria, and dye-related discoloration, which should be clinically distinguished [3,5]. Awareness of PUBS is essential to differentiate it from hematuria, porphyria, or myoglobinuria and to prevent unnecessary anxiety. In our patient, advanced age, CKD, and alkaline urine were key predisposing factors. Metabolic disturbances such as hyperglycemia and reduced renal clearance may alter urinary substrate composition, facilitating bacterial colonization and pigment production, while impaired host defenses in chronic disease states may further contribute to bacterial persistence. Conservative management and observation without antibiotic therapy led to complete recovery. Recognizing PUBS in home-based catheter care settings prevents unnecessary emergency admissions and reduces inappropriate antimicrobial prescribing.

## Conclusions

PUBS is a visually alarming but generally benign and asymptomatic manifestation in catheterized elderly patients. Prompt recognition, catheter replacement, adequate hydration, and conservative management are usually sufficient. Awareness of this phenomenon helps clinicians reassure caregivers and avoid overtreatment. Clinicians should also consider underlying metabolic or renal factors that predispose to alkaline urine and pigment formation. Regular catheter care and patient education can further reduce recurrence and anxiety associated with this condition. Maintaining adequate hydration along with proper catheter care remains a key preventive strategy for PUBS.
